# Do school crossing guards make crossing roads safer? A quasi-experimental study of pedestrian-motor vehicle collisions in Toronto, Canada

**DOI:** 10.1186/s12889-015-2065-y

**Published:** 2015-07-31

**Authors:** Linda Rothman, Daniel Perry, Ron Buliung, Colin Macarthur, Teresa To, Alison Macpherson, Kristian Larsen, Andrew Howard

**Affiliations:** Child Health Evaluative Sciences, Toronto, Canada; Orthopaedic Surgery, The Hospital for Sick Children, Toronto, Canada; Faculty of Health-School of Kinesiology & Health Science, York University, Toronto, Canada; Division of Health Sciences, Warwick Medical School, Warwick University, Coventry, UK; Department of Geography, University of Toronto Mississauga, Mississauga, Canada; Department of Pediatrics, University of Toronto, Toronto, Canada; Department of Surgery, University of Toronto, Toronto, Canada; Health Policy Management and Evaluation, University of Toronto, Toronto, Canada; Dalla Lana School of Public Health, University of Toronto, Toronto, Canada

**Keywords:** Motor vehicles, Walking, Injuries, Public health, Prevention, Schools

## Abstract

**Background:**

The presence of school crossing guards has been associated with more walking and more pedestrian-motor vehicle collisions (PMVCs) in area-level cross-sectional analyses. The objectives of the study were to (1) Determine the effect on PMVC rates of newly implemented crossing guards in Toronto, Canada (2) Determine where collisions were located in relation to crossing guards throughout the city, and whether they occurred during school travel times.

**Methods:**

School crossing guards with 50 m buffers were mapped along with police-reported child PMVCs from 2000–2011. (1) A quasi-experimental study identified all age collision counts near newly implemented guards before and after implementation, modeled using repeated measures Poisson regression adjusted for season and built environment variables. (2) A retrospective cohort study of all child PMVCS throughout the city to determine the proportions of child PMVCs which occurred during school travel times and at guard locations.

**Results:**

There were 27,827 PMVCs, with 260 PMVCs at the locations of 58 newly implemented guards. Repeated measures adjusted Poisson regression found PMVCs rates remained unchanged at guard locations after implementation (IRR 1.02, 95 % CI 0.74, 1.39). There were 568 guards citywide with 1850 child PMVCs that occurred at guard locations. The majority of child PMVCs occurred outside school travel times (*n* = 1155, 62 %) and of those that occurred during school travel times, only 95 (13.7 %) were at a guard location.

**Conclusions:**

School crossing guards are a simple roadway modification to increase walking to school without apparent detrimental safety effects. Other more permanent interventions are necessary to address the frequency of child PMVCs occurring away from the location of crossing guards, and outside of school travel times.

## Background

There is a worldwide epidemic of trauma, accounting for 5.8 million global deaths each year [1]. Approximately 4000 deaths each day are directly attributable to road traffic collisions [2]. Most fatal road crashes occur in low and middle income countries, however the problem transcends race and income, such that in high income countries road traffic collisions are a leading cause of death amongst children and adolescents [3–5]. In 2012, road traffic collisions resulted in 278 child deaths (≤19) and almost 26,000 injuries in Canada [6].

Child pedestrians account for 22 % of child road traffic fatalities [7]. A large proportion of ‘exposure’ as a pedestrian is amassed during the narrow time-period defined by the journey to school [8–10]. The long-term health benefits of physical activity through walking to school are widely promoted via national and international campaigns [11–14], with approximately 50 % of children in Ontario opting to walk to school [11, 15, 16]. It is therefore, unsurprising that a study from Toronto in Ontario, Canada demonstrated that almost 50 % of child pedestrian- motor vehicle collisions (PMVCs) (<18 years) occurred during school travel times, and that over one third occurred within 300 m of a school [17]. Whilst walking to school may have many health benefits, there are therefore inherent risks that must be addressed; primarily related to pedestrian exposure to road traffic.

The pedestrian environment may be modified in a number of ways to promote safe pedestrian travel, which include speed restrictions, pedestrian-only areas, traffic signals, and adult crossing guards. It has been demonstrated that an increased presence of road safety measures generally increases the number of children walking to school [18], and there is specific evidence from qualitative parental interviews and cross-sectional observational studies that the presence of a crossing guard increases pedestrian journeys to school [19, 20], although some debate exists [21]. This suggests that there is a widespread assumption amongst parents, and the wider public, that crossing guards have a favorable influence on traffic behaviour, and on pedestrian deaths, though this notion has largely been unchallenged. Recent evidence has emerged to suggest that crossing guards may be associated with more PMVCs in area level analyses [22, 23], however it was unclear if the presence of crossing guards were markers for particularly dangerous locations, or if cross-sectional data masked the effect of the guards.

This study seeks to determine the effect of newly implemented crossing guards on PMVC rates. This study will also assess where school crossing guards were located city-wide in relation to child PMVCs and the proportion of PMVCs that occurred during school travel times.

## Methods

**Fig. 1:**
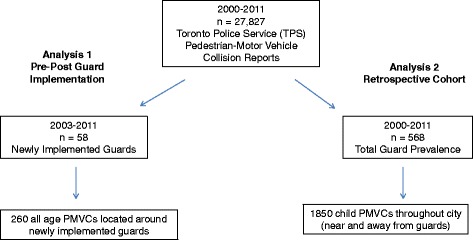
Flowchart of Study Analyses

The study was conducted in Toronto, Canada. Two related analyses are presented, using collision data extracted from routinely collected Toronto Police Service pedestrian-motor vehicle collision reports from 2000–2011 (Fig. 1). Police-reported pedestrian-motor vehicle collisions include those resulting in no injury, minimal injury, minor injury (seen in the emergency department), major injury (admitted to hospital), and fatal injury. The location of the incident was provided as longitudinal and latitudinal coordinates. Each collision record included the date and time of the collision, and the age of the pedestrian(s) involved in the collisions.

The first analysis used a quasi-experimental design to determine the effects of newly implemented school crossing guards. The Toronto Police Service supplied details of all newly implemented crossing guards during the period 2003 – 2011. The information included the implementation date, the intersections at which all crossing guards were located and the school crossing guard duty time. When time was not specifically indicated, duty times were assumed to be the standard guard contracted times across the city; 08:00 – 09:00, 11:30 – 1:00 and 3:00 – 4:15 on weekdays only. Major school holidays across Toronto for the study period were determined from the Toronto District School Board website, and these were identified as non-duty times.

The analysis focused only on collisions from 2000–2011 which occurred at locations where new guards were implemented from 2003–2011 (Fig. 1). The outcome of interest was the collision count for all ages within 50 m of the newly implemented guard location. The independent variable was a dichotomous variable, designated as either pre or post guard implementation. Covariates included a dichotomous winter variable (October– March), and several built environment features measured at the school crossing guard location. Road type at the crossing location was provided by the City of Toronto, and was included as a categorical variable (Midblock location: collector road, local road, major road; Intersection location: local/local, major/major, major/local, collector/collector and collector/local). The age of the neighborhood was calculated based on the era of development from the 2006 Census; this binary variable was based on >50 % of neighborhood constructed before or after 1960 (0 = majority of neighborhood constructed after 1960, 1 = majority of neighborhood constructed prior to 1960). The demarcation between pre and post 1960 was used as it reflected the different characteristics of school neighbourhoods where crossing guards are located in Toronto. In the Canadian context, typical modern suburbs developed during the period of urban renewal that occurred in many cities in the 1960s. Most of the neighbourhoods constructed before 1960 are within the older central city core of Toronto. A land use mix variable was also constructed based on parcel level data from the Municipal Property Assessment Corporation (MPAC). Land use mix was calculated based on a 1000 m straight line buffer from the guards location (measured using an entropy index where scores of 0 = single land use, 1 = equal distribution of all land use classifications, i.e. residential, commercial, institutional, industrial, recreational/open space) [24, 25].

The second analysis involved a retrospective cohort formed from all child PMVCs, ages 4–12 from 2000–2011 throughout Toronto (Fig. 1). The analysis was conducted to assess where all school crossing guards were located (i.e. not just the ones where implementation dates were available) in relation to child PMVCs and the proportions of these collisions which occurred during school travel times. The Toronto Police Service supplied the location of all crossing guards in Toronto for the years 2010–11, which was assumed to be a consistent location over the collision study period. A bivariate variable was created indicating whether the collision occurred during school travel times as defined in the first analysis (08:00 – 09:00, 11:30 – 1:00 and 3:00 – 16:15 on weekdays only) and excluded major school holidays. Implementation dates were not available for all of these guards and therefore, could not be included in the quasi-experimental analysis However, these data provided some descriptive information regarding the location of child PMVCs specifically (i.e. near or far from locations identified as needing school crossing guards) and school travel times throughout the city.

### Statistical analysis

The unit of analysis was the crossing guard location, with the location of guards and PMVCs mapped using ArcGIS (ESRI: ArcGIS, version 10). A straight line (Euclidean) distance buffer of 50 m was created around crossing guards to assess the proportion of PMVCs occurring within the vicinity of a crossing guard. The 50 m buffer was selected to locate the collision as close as possible to the crossing guard, while still maintaining a meaningful number of collisions and allowing for some minor collision location error. The recorded PMVC locations were superimposed and the collision counts, with reference to the 50 m buffers, were determined. Statistical analysis was conducted using SAS (SAS, version 9.3).

#### Analysis 1: Newly implemented school crossing guards (2003–2011)

The period at risk, i.e. number of months of exposure pre and post crossing guard implementation was determined, and the number of all PMVCs per month/school crossing guard with reference to the 50 m buffer, was calculated. Descriptive statistics were conducted of the PMVCs occurring within the 50 m buffer of the crossing guards. Repeated measures Poisson regression was used to model counts of PMVCs per school crossing guard month (subsequently referred to as SCG month) pre and post intervention, which controlled for winter and environmental features and accounted for repeated measures of PMVCs around each crossing guard location. Over-dispersion of the data was tested and was not found to be present in these data. The offset used in the regression analysis was calculated as time per school crossing guard-month, as pedestrian volume data was unavailable. Model-based standard error estimates were used, due to the small sample size [26]. Incident rate ratios ((IRR with 95 % confidence intervals (CI)) were calculated by exponentiating the beta coefficients from the regression models. The model was rerun for just collisions that occurred during times that school crossing guard duty time.

#### Analysis 2: All City of Toronto school crossing guards (2010–2011)

The proportions of PMVCs which occurred at locations where school crossing guards were implemented and during school travel times hours were calculated for children (4–12 years).

## Results

There were a total of 27,827 pedestrian-motor vehicle collisions reported to the Toronto police from 2000–2011.

### Analysis 1: Newly implemented school crossing guards (2003–2011)

**Fig. 2:**
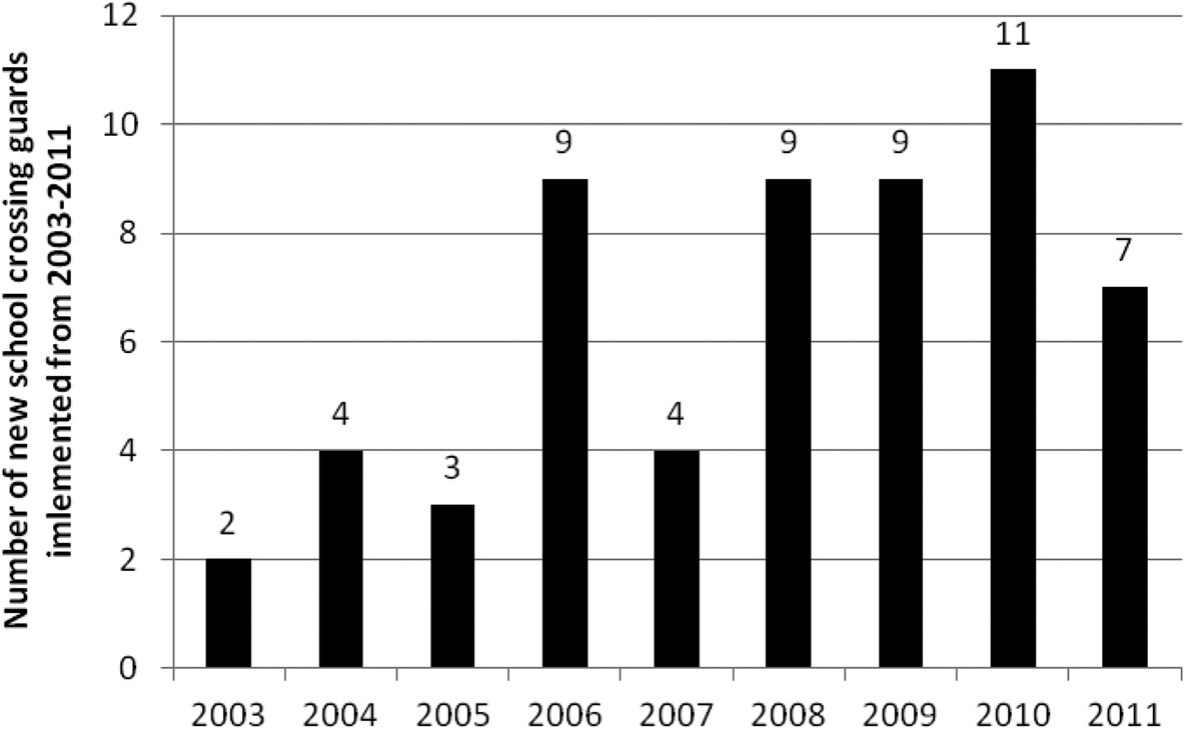
Number of new school crossing guards implemented by year

There were 58 new crossing guards for which implementation dates were available from 2003–2011 which were included in the analysis. The majority having been implemented from 2008 onwards (Fig. 2). The majority of the guards were located at intersections.

There were 260 PMVCs that occurred within 50 m of these crossing guards. The majority of the PMVCs occurred in the adult age group (19 – 64 years), followed by older adults (65 years +) and then by elementary school age children (4 – 12 years, Table 1). Seventy (27 %), of the PMVCs involved children < 19 years of age. Almost 60 % of PMVCs resulted in an emergency department visit or hospital admission. There were two adult pedestrian fatalities near crossing guard locations during this time period, with 337 adult fatalities across the entire city. Seventy-nine PMVCs (30 %) occurred during crossing guard duty times and 181 (70 %) occurred outside of duty times.

**Table 1:** Descriptive statistics of PMVC occurring near newly implemented school crossing guards (*n* = 260)

Variable	N (%)
Age	
Infant (0–4)	4 (1.6 %)
Elementary age (4–12)	31 (12.5 %)
Middle school age (13–14)	16 (6.5 %)
High school age (15–18)	19 (7.7 %)
Adult (19–64)	142 (57.3 %)
Older adult (65+)	36 (14.5 %)
Injury Severity	
No injury	22 (8.5 %)
Minimal	82 (31.5 %)
Minor (seen in emergency department)	136 (52.3 %)
Major (admitted to hospital)	18 (6.9 %)
Fatal	2 (0.8 %)
Crossing guard duty hours	
During duty hours	79 (30.4 %)
Outside of duty hours	181 (69.6 %)

There were 5826 crossing-guard months prior to the implementation of school crossing guards with a total of 176 PMVCs (crude rate of 3/100 cross-guard months), and 2584 months of exposure following implementation with a total of 84 PMVCs (crude rate of 3.3/100 crossing-guard months). The majority of the PMVCs occurred during the winter months (158, 61 %, Table 2). Fifty two guards (90 %) were located at intersections with almost 60 % of guards located at either collector/local road or major/major road intersections. The six guards located at midblock locations on collector roads were in front of schools and a public library. There were no new school crossing guards implemented at local road, major road midblock locations, or at collector/major intersection locations during the study period. Nineteen (33 %) of the guards were located in a neighbourhood that was constructed prior to 1960. The average entropy index land use score was 0.71, indicating a moderately high level of land use mix near school crossing guards.

**Table 2:** Frequency and adjusted incidence rate ratios with 95 % confidence intervals of PMVC by implementation and season and crossing guard location characteristics

Variable	N (%)	Adjusted IRR (95 % CI)
Collision characteristics:		
School crossing guard implementation		
Pre implementation	176	1.00
Post implementation	84	1.02 (0.74, 1.40)
Season		
Non-winter	102 (39.2 %)	1.00
Winter	158 (60.8 %)	1.56 (1.15, 2.11)
School Crossing guard location characteristics:		
Road type		
Intersections:		
Major/major	15 (26 %)	1.00
Local/local	7 (12 %)	0.03 (0.01, 0.15)
Major/local	9 (16 %)	0.42 (0.29, 0.62)
Collector/collector	2 (4 %)	0.03 (0.00, 0.29)
Collector/local	19 (33 %)	0.06 (0.03, 0.12)
Midblock locations:		
Collector	6 (10 %)	0.13 (0.06, 0.28)
Neighbourhood age		
Post 1960 neighbourhood	39 (66 %)	1.00
Pre 1960	19 (33 %)	0.46 (0.31, 0.69)
Land use mix		
Mean Entropy score	0.71 (SD ± 0.14)	16.11 (5.00, 52.07)

No significant change in collision risk was evident following the implementation of a crossing guard (IRR 1.02, 95 % CI 0.74, 1.40, Table 2). More PMVCs were associated with winter months (IRR 1.56, 95 % CI 1.15, 2.11). All road types had significantly lower rates of PMVC compared to crossing guard locations at major/major roadway intersections. Neighbourhoods with a higher proportion of housing built before 1960 (IRR 0.46 95 % CI 0.31, 0.69) were also associated with fewer PMVCs. More PMVCs were associated with greater land use mix (IRR 16.11, 95 % CI 5.00, 52.07)); however, this result should be interpreted with caution due to low estimate precision as a result of high variability in the data.

Similarly, there was no significant change in collision risk evident when the model was run only for the 79 collisions that occurred during school crossing guard duty times (results not shown, IRR, 1.25, 0.80, 1.95). Road type could not however, be controlled for in this analysis due to instability of the estimates as a result of low numbers.

### Analysis 2: All City of Toronto school crossing guards (2010/2011)

There were 568 school crossing guards and 1850 PMVCs involving children ages 4–12 during the study period throughout Toronto. The majority of child PMVCs occurred outside school travel times (*n* = 1155, 62 %, Table 3). Of the 695 child PMVCs that occurred during school travel times, only 95 (13.7 %) occurred at a location where crossing guards were implemented.

**Table 3:** Proportions of child PMVCs occurring near (within a 50 m buffer) and distant to a school crossing guard (SCG) location during school and non-school travel times

	Non-school travel time			School travel time		
	N	Near SCG location	Distant to SCG location	N	Near SCG location	Distant to SCG location
Children (4 – 12)	1155	138 (12 %)	1017 (88 %)	695	95 (13.7 %)	600 (86.3 %)

*Abbreviations*: CI confidence intervals; IRR incident rate ratios; PMVC pedestrian-motor vehicle collision; SCG school crossing guard

## Discussion

A total of 27,827 PMVCs occurred in the City of Toronto from 2000–2011, with 2573 occurring at school crossing guard locations. There was no significant change in collision rates with the implementation of new school crossing guards after controlling for time, season and the built environment. The majority of children’s PMVCs occurred outside of school travel times and distant from crossing guard locations throughout the city.

According to Toronto Police Services, school crossing guards are most likely to be implemented in locations, particularly intersections, with higher traffic risks due to characteristics of the roadway and/or large numbers of children crossing [27]. It is possible that the effect of the guards was not strong enough to overcome this increased danger. It is also possible that the null result may actually be indicative of a positive safety effect of the guards, as pedestrian volumes are likely to increase at a specific location with the implementation of a school crossing guard; thereby increasing pedestrian exposure to traffic. Unfortunately, one of the limitations of the study was that it was not possible to incorporate either traffic volumes or walking exposure in these analyses, as traffic and pedestrian volume data is not routinely collected in Toronto on smaller neighbourhood roads. Previous work found an association between higher proportions of children walking to school and the presence of a guard in Toronto [20]. School travel times also correspond with highest vehicle and pedestrian traffic times and increased exposure of pedestrians to vehicular traffic [28]. Vehicle volumes have increased in the City of Toronto over the last 10 years [29] and higher vehicle volumes are known to be associated with higher rates of PMVCs [28, 30–32]. Therefore, if it were possible to incorporate pedestrian volume in these analyses, a lower rate of collision per pedestrian crossing may have been evident. A future study is planned to compare pedestrian counts obtained by the Toronto Police during the crossing guard eligibility site survey, to those obtained after the guard is implemented, in order to control for the differences in exposure.

An important finding was that only 38 % of PMVCs involving elementary school aged children occurred within school travel times and only 5 % occurred near locations where crossing guards were implemented. This implies that the majority of PMVCs involving children cannot be targeted using existing school crossing guards and other interventions must be directed towards reducing PMVCs involving children outside of school travel times. Furthermore, even during school travel times, it appears that there are insufficient numbers of guards to address the scope of the child pedestrian problem, as the vast majority of school travel time PMVCs occurred outside the vicinity of a school crossing guard location.

The first analysis of new crossing guards was limited by the small size of the study as implementation dates of school crossing guards were only available back to 2003 and due to the infrequency of the collision outcome. This resulted in some unstable estimates when only including PMVCS that occurred only during school crossing guard duty times; however, it appeared that the null effect of the guards was maintained during guard duty times. The small numbers also made it prohibitive to restrict the sample to just children. It is felt that it was reasonable to include all ages in the analysis, as the duties of crossing guards in Toronto include assisting with crossing of all people regardless of age, at the location. As mentioned previously, it was also not possible to account for pedestrian exposure. Observed walking to school counts have been used previously as proxies for exposure to traffic in children in Toronto, and a strong positive association between a school crossing guard and walking to school has been found [20]. In addition, walking rates did not drive child PMVC rates once the built environment was modeled [23]. Traffic volume could also not be accounted for due to lack of availability of accurate data. Finally, other measures to increase road safety were not considered within these analyses; therefore, if the implementation of a crossing guard were part of a broad risk reduction strategy, the guard could potentially act as a confounding variable for another traffic control measure. Personal discussion with officers from the Toronto Police Services (TPS) indicated that school crossing guards are usually associated with new road furniture (i.e. signage detailing the presence of a school crossing guard). (Personal communication, 2014, TPS) which may have an independent effect beyond simply the presence of the crossing guard.

Strengths of this study included the pre-post design in the first analysis, therefore avoiding potential bias due to temporal and seasonal effects. The repeated measures design has the added benefit of controlling for other potentially confounding factors related to the environment not considered assuming these remained unchanged over the study period. The large population-based sample of the second analysis also allowed for generalizability of the results throughout the City of Toronto.

## Conclusions

There was no increase in collision rates after the implementation of school crossing guards, despite previous evidence suggesting an association between higher collision rates, higher walking rates and the presence of crossing guards. Crossing guards therefore, appear to be a simple roadway modification particularly at intersections, to increase walking to school which is not associated with an increase in PMVCs. Crossing guards may have an overall positive effect on PMVCs, although this is not demonstrable without measurement of walking exposure. These results emphasize the need to develop methods to measure both walking exposure and traffic volumes in order to accurately assess traffic control interventions. Finally, more definitive interventions are necessary to address the high burden of child PMVCs that occur outside school travel times, and beyond the boundaries of crossing guards.

### Availability of supporting data

Interested parties may contact the corresponding author regarding data requests.
